# Significance of exosomes in osteosarcoma research: a systematic review and meta-analysis of a singular clinical investigation

**DOI:** 10.3389/fcell.2024.1473044

**Published:** 2024-11-13

**Authors:** Xuehong Liu, Jingyao Ye, Wenlong Guo, Junqing Wang

**Affiliations:** ^1^ Institute of Integrated Traditional Chinese and Western Medicine, Chinese Evidence-based Medicine Center, West China Hospital, Sichuan University, Chengdu, China; ^2^ Department of Orthopedics, Hospital of Chengdu University of Traditional Chinese Medicine, Chengdu, China; ^3^ Clinical School of Medicine, Chengdu University of Traditional Chinese Medicine, Chengdu, China; ^4^ Department of Orthopedics, The Second Affiliated Hospital of Shandong University of Traditional Chinese Medicine, Jinan, China

**Keywords:** osteosarcoma, exosomes, preclinical studies, meta-analysis, chemotherapy

## Abstract

**Background:**

Osteosarcoma is the most prevalent among primary bone malignancies, and its standard intervention involves neoadjuvant chemotherapy - surgical adjuvant chemotherapy (MAP regimen) with adriamycin, cisplatin, and high-dose methotrexate. Early-stage osteosarcoma can be effectively treated with surgical resection along with chemotherapy or radiotherapy. However, as the cancer progresses, the efficacy of chemo- and radiotherapy decreases, and the associated problems increase. The current understanding of osteosarcoma development, diagnosis, and treatment does not meet clinical demands. More recently, there has been a significant increase in exosome-associated osteosarcoma research, potentially opening up novel possibilities for osteosarcoma research.

**Purpose:**

We comprehensively evaluated and analyzed the advancement of preclinical research related to exosome-osteosarcoma. We aimed to establish a practical, theoretical foundation for future research initiatives.

**Study design:**

The selected design was a systematic review and meta-analysis.

**Methods:**

Scientific databases, such as PubMed, Embase, The Cochrane Library, and Web of Science, were extensively screened for exosome and osteosarcoma articles. Two highly trained investigators separately reviewed the literature, extracted relevant information, and assessed study quality. Subsequently, we conducted a meta-analysis using Review Manager 5.4.

**Results:**

In total, 25 animal-based randomized controlled trials (RCTs) were selected for analysis. Among them, 13 studies provided strong evidence of cellular exosomes regulating osteosarcoma development from bone marrow mesenchymal stem cells, osteosarcoma cells, and macrophages. In addition, 12 studies demonstrated the therapeutic potential of exosomes in managing osteosarcoma, among which 7 studies transplanted transfected exosomes directly into animals as drugs, and five studies employed exosomes as drug carriers, which were next transplanted into animals.

**Conclusion:**

Based on our meta-analysis, macrophages strongly modulate osteosarcoma development, and engineered exosomes provide the most effective exosome-based osteosarcoma treatment.

## 1 Introduction

Osteosarcoma ranks first among all bone-related primary malignant tumors, with a reported incidence of about 4.8 per million, mainly in children and adolescents (Pingping et al., 2019; Lancia et al., 2019), and third among all cancers among cancers among children aged <20 years (Belayneh et al., 2021; Simpson and Brown, 2018). Statistically, approximately 2% of all tumors among children <14 years of age and 3% among children between 14–19 years is osteosarcoma (Siegel et al., 2022; Mirabello et al., 2009). Owing to its mesenchymal tissue origin, Osteosarcoma features malignant spindle cells that produce aberrant bone-like tissue (Ji et al., 2015). There are multiple reported risk factors for osteosarcoma, including high stature, elevated birth weight, certain inherited cancer susceptibility syndromes, and common genetic variants (Gianferante et al., 2017). At present, the optimal standard of care is neoadjuvant chemotherapy - surgical adjuvant chemotherapy with adriamycin, cisplatin, and high-dose methotrexate (MAP regimen) (Ritter and Bielack, 2010). This management is efficacious for early-stage osteosarcoma. However, the chemo- and radiotherapeutic efficacies decline with disease progression (Smrke et al., 2021). Moreover, conventional chemotherapeutic agents, namely, methotrexate, cisplatin, adriamycin, and iso-cyclophosphamide, which are often employed in osteosarcoma management, are accompanied by numerous complications, such as unstable blood levels, rapid clearance, poor targeting, variable toxicity to normal cells, and drug resistance. As such, their application among osteosarcoma patients is restricted (Grünewald et al., 2020). Given these factors, the past few decades have seen no marked improvement in patient survival (Corre et al., 2020). Post-recurrence patient prognosis also remains poor, with only 23%–29% of patients surviving over 5 years following a second diagnosis (Simpson and Brown, 2018) and long-term survival being <20% (Carrle and Bielack, 2006; Ferrari et al., 2003). The consequences of osteosarcoma can be utterly devastating, affecting both the physical and mental health of patients and their families (Mortus et al., 2014). Therefore, a safe and effective therapy is urgently needed for appropriate osteosarcoma management.

Exosomes are nanoscale extracellular vesicles that are released by a wide variety of cells (Liu et al., 2021a). Its primary role is normal cellular metabolic maintenance and intercellular communication (Song et al., 2021). Based on transmission electron microscopy images, exosomes resemble “cups” or “discs,” which are molecularly recognized by expressing specific protein markers. These structures mediate cell-to-cell communication via the transfer of reactive molecules, namely, proteins, DNA, mRNAs, and non-coding RNAs, from 1 cell to another (Tkach and Théry, 2016). Exosomes are made up of lipid bilayers. Hence, they are readily taken up by neighboring or distant receptor cells. Exosomal contents (such as small RNAs and proteins) provide physiological activities, such as immunomodulation (Teng et al., 2015), autophagy (Baixauli et al., 2014), stem cell differentiation (Nair et al., 2014), and intercellular communication (Tkach and Théry, 2016), in a process termed the third mode of cellular communication. There is significant interest in the development of exosomes for the management of osteosarcoma. The Exosomal lipid barrier protects exosomal miRNAs from RNase degradation, enabling stable transfer between two separate tumors and involvement in tumor growth (Tkach and Théry, 2016). Exosomal miRNAs significantly influence the cellular capacities for migration, proliferation, invasion, apoptosis, and chemoresistance in many cancers, all closely associated with patient prognosis, including osteosarcoma patients (Santos et al., 2018). At present, researchers believe that exosomes have broad prospects for treating osteosarcoma (Zhongyu et al., 2024). Exosomes are crucial for the onset and progression of osteosarcoma. Exosomes can increase osteosarcoma lung metastasis and negatively impact prognosis by modulating MMP and other processes that alter the interstitial microenvironment (Zhan et al., 2022). Conversely, exosomes are also crucial for osteosarcoma’s drug resistance. Osteosarcoma cells produce tumor cells resistant to cisplatin by activating autophagy, breaking down cell components, and preserving intracellular balance through exosomes. However, the role of exosomes not only worsens the disease but also plays an advantage in treatment. For example, exosomes of sensitive cells treated with Luteolin can improve the adriamycin response in adriamycin-resistant cells by increasing miR-384 and targeting the PTN/β-catenin/MDR1 axis. It is a promising therapeutic agent for chemotherapy-resistant OS (Qin et al., 2022). As a result, exosomes may be considered a double-edged sword in regulating bone tumor formation, influencing carcinogenesis, angiogenesis, and metastasis while inhibiting tumor progression (Vakhshiteh et al., 2019). Therefore, investigating the role of exosomes in the progression of osteosarcoma is essential.

## 2 Materials and methods

The analysis and findings were reported following the Preferred Reporting Items for Systematic Reviews and Meta-Analyses (PRISMA) guidelines (Liberati et al., 2009). This study did not require patient permission and ethical approval because the data and results were derived from previously approved research. The protocol was entered into the PROSPERO database (CRD42024502574).

### 2.1 Inclusion criteria

#### 2.1.1 Patients and diseases (P)

Any animal and osteosarcoma cell lines used to produce osteosarcoma xenograft models were included.

#### 2.1.2 Intervention (I)

Collect any interventions involving extracellular vesicles that inhibit the development of animal osteosarcoma models.

#### 2.1.3 Control (C)

Positive control: exosomes from various transfected cells or drug-carrying exosomes.

Negative control: blank, DMEM, PBS, saline.

#### 2.1.4 Results (O)

Tumor weight (mg).

Tumor volume (mm3) (tumor volume = length × width2/2) (Li et al., 2024)

#### 2.1.5 Type of study (S)

Control studies were included, with no restrictions on the blind method.

### 2.2 Exclusion criteria

1) Irrelevant animals: Animals with comorbidities; non-mouse animal model studies; *ex vivo*, *in vitro*, and computer models.
2) Irrelevant controls: No controls for osteosarcoma.
3) Irrelevant study types: Case, crossover, and studies without independent control groups.
4) Unavailable data: specific study details or study data (e.g., reviews or conference abstracts) were missing.
5) Unreported studies of (1) tumor weight; (2) tumor volume.
6) Non-English studies.
7) Non-peer-reviewed journal articles

### 2.3 Data selection

Owing to the diverse collection of contemporary preclinical studies involving animal models as test subjects, we analyzed data involving exosomes promoting osteosarcoma development and suppressing osteosarcoma progression. Regarding exosomes promoting osteosarcoma development, we generated subcategories based on varying cellular sources of exosomes. In the case of exosome suppression osteosarcoma progression, we generated subcategories based on the varying mechanisms of exosomal action.

### 2.4 Data source and screening

Relevant articles were selected via extensive screening of four major scientific databases, PubMed, Embase, Web of Science, and Cochrane, published from 2014.03.01 to 2024.03.01. Screening was completed using a combination of the following terminology: [(“Exosomes” OR “Extracellular Vesicles” OR “Extracellular Vesicle” OR “Vesicle, Vesicle. Extracellular” OR “Vesicles, Extracellular” OR “Exovesicles” OR “Exovesicle”) AND (Osteosarcoma OR Osteosarcomas OR “Osteosarcoma Tumor” OR “Osteosarcoma Tumors” OR “Tumor, Osteosarcoma” OR “Tumors,” Osteosarcoma’ OR “Sarcoma, Osteogenic” OR “Osteogenic Sarcomas” OR “Sarcomas, Osteogenic” OR “Osteogenic Sarcoma” OR “Sarcoma, Ewing” OR “Sarcoma, Ewing’s” OR “Sarcoma, Ewings” OR “Ewing’s Sarcoma” OR “Ewings Sarcoma” OR “Ewing’s Tumor” OR “Ewings Tumor” OR “Tumor, Ewing’s” OR “Ewing Sarcoma” OR “Ewing Tumor” OR “Tumor, Ewing” OR “Osteosarcoma, Juxtacortical” OR “Juxtacortical Osteosarcoma” OR “Juxtacortical Osteosarcomas’ OR ‘Osteosarcomas. Juxtacortical”)]

### 2.5 Literature screening and data acquisition

After removing duplicate studies, two highly trained investigators reviewed the eligible articles and extracted data based on the inclusion/exclusion criteria, cross-checking their selections. Any disagreements were resolved by a third investigator. Data acquisition was based on the pre-established full-text data sheets, which included (1) basic study characteristics: article title, authors, publication year, type of study, baseline features of mice, sample size, modeling method, exosome cell type and source, exosome size, and marker proteins; (2) critical elements of the bias risk assessment; and (3) outcome measures of tumor volume and weight. In the case of studies reporting data in image form only, we employed the GetData Graph Digitizer software for data extraction from images.

### 2.6 Bias risk between included studies

Two highly trained investigators evaluated and cross-checked the inherent bias risk of the selected studies based on the SYRCLE’s Risk of Bias in Animal Studies tool (Hooijmans et al., 2014). They covered selection bias, implementation bias, measurement bias, follow-up bias, reporting bias, and any other bias as part of a list of 10 questions or tools. Any disagreements were resolved via discussion with a third investigator. The evaluation questions were answered either with a “yes” (indicating low bias risk) or “no” (indicating high bias risk). Unclear items were classified as “not clear.”

### 2.7 Data analyses

Among the extracted data were tumor volume and weight. All data were analyzed using Review Manager 5.4 software. We conducted a sensitivity analysis to determine if excluding studies with a high risk of bias affected the estimated effect or heterogeneity of the outcome. Forest plots are depicted aggregated hazard ratios with their respective 95% confidence intervals. The inconsistency index (I^2^) was employed to assess statistical heterogeneity. Data exhibiting greater heterogeneity were analyzed using a random-effects model, while data demonstrating lesser heterogeneity were evaluated using a fixed-effects model. To address the uniqueness of animal research and to reduce bias from varying experimental designs, we select to use the mean difference to convey the impact of the intervention modality on the outcome measures. Std. Mean difference (SMD) is the quotient between the difference of two means and the combined standard deviation. It eliminates the influence of absolute value and measurement unit of a study, and is suitable for the analysis of numerical data with different units or large difference in means.

## 3 Results

### 3.1 Systematic screening

**FIGURE 1:**
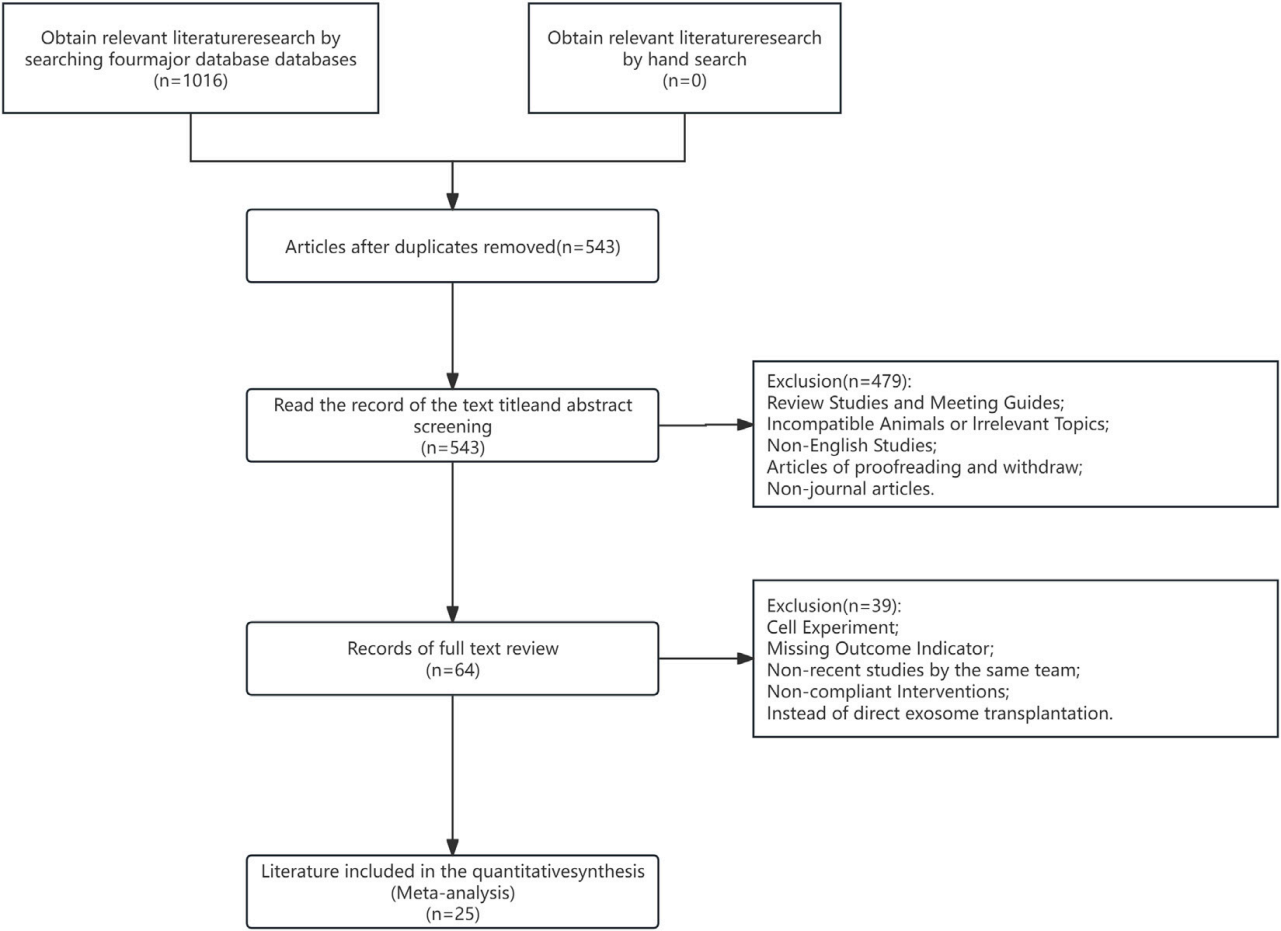
Flow chart of article selection.

Overall, 1,016 articles were eligible for analysis. Among them, duplicate and irrelevant articles (e.g., subject inconsistency, reporting, *etc.*) were eliminated after reviewing study titles and abstracts. Subsequently, articles with inconsistent research methods (e.g., cellular experiments, endpoint indicators without tumor volume or tumor weight, and so on) were eliminated following the full-text review. Lastly, 25 preclinical studies were selected for meta-analysis. A PRISMA flowchart depicting our strict study inclusion criteria is provided in Figure 1.

### 3.2 Basic demographics of included studies

All selected studies were randomized controlled trials (RCTs), with the mouse as the examined animal. Overall, six studies failed to report the sex of mice. The age of model mice ranged between 3–8 weeks, and the sample size was between 10–68 mice. The model type was inoculated cells. Exosomes were derived from humans (17 studies), other sources (6 studies), and source unspecified (2 studies). Exosomal cell types were osteosarcoma cells (5 studies), bone marrow mesenchymal stem cells (12 studies), macrophages (3 studies), and other cells (5 studies). The exosomal diameter was between 30–200 nm. The exosome-specific surface proteins assessed were CD9, CD29, CD34, CD44, CD63, CD 81, CD105, CD200, HSP 70, Hsp 90, TSG101, and Alix. The information mentioned above is detailed in Tables 1, 2.

**TABLE 1:** Characteristics of included studies (1).

No.	Study	Year	Species	Number	Age(w)	Sex	Model	Source	Type	Size (nm)	Protein
1	Lin et al. (2019)	2018	BALB/c nude mice	24	5	male	U2OS/NC cell	—	BMSC	—	CD29,CD44
2	Zhao et al. (2019)	2019	BALB/c nude mice	36	6–8	female	MNNG/HOS cell	human	BMSC	40–150	CD81,CD63
3	Li et al. (2021a)	2021	BALB/c nude mice	40	5–6	male	HOS cell	human	Osteosarcoma cell	40–200	CD63,CD81,TSG101
4	Zhang et al. (2021)	2021	C57/BL/6 mice	—	6	male	C3H cell	human	Osteosarcoma cell	100	Hsp70,Hsp90,CD63,CD81
5	Li et al. (2021b)	2021	nude mice	20	4	—	MG-63 cell	mice	BMSC	100–200	HSP70,CD9
6	Qi et al. (2021)	2021	nude mice	—	—	—	MG-63 cell	human	BMSC	30–165	CD63
7	Shi et al. (2021)	2021	BALB/c nude mice	24	4–6	male	MG-63 cell	human	BMSC	30–120	ALIX,CD81,TSG101
8	Zhang et al. (2021)	2021	BALB/c nude mice	22	—	—	143B cell	human	macrophage	100	CD9,CD63
9	Liu et al. (2021b)	2021	BALB/c mice	20	4–8	male	143B/Saos2 cell	human	macrophage	30–130	CD63,CD9,Tsg101
10	Zhan et al. (2022)	2022	BALB/c nude mice	—	6	female	MG-63 cell	human	Osteosarcoma cell	30–150	CD63,CD9
11	Zhu et al. (2022)	2022	BALB/c nude mice	—	4–6	female	143 B cell	human	BMSC	40–100	CD9,CD63,CD81
12	Feng et al. (2022)	2022	BALB/c nude mice	48	4	—	Saos2 cell	mice	BMSC	100	CD63,CD81,TSG101
13	Yan et al. (2023)	2023	nude mice	—	—	—	MG-63 cell	human	macrophage	30–150	CD34,CD81,CD44,CD200,CD105

**TABLE 2:** Characteristics of included studies (2).

No.	Study	Year	Species	Number	Age(w)	Sex	Model	Source	Type	Size (nm)	Protein
1	Xu et al. (2020b)	2020	BALB/c nude mice	10	4	male	HOScell	human	BMSC	60–100	CD9,CD63
2	Lu et al. (2022)	2021	BALB/c nude mice	—	3–4	female	MG-63cell	human	cartilage cell	100	CD63,CD81
3	Keremu et al. (2022)	2022	athymic nude mice	36	6	female	143Bcell	human	BMSC	60–160	CD9,CD63,CD81
4	Huang et al. (2022)	2022	Nude mice (BALB/c)	—	4–5	female	MNNG/HOScell	human	osteosarcoma cell	132.5 ± 12.9	HSP70,CD63,CD81
5	Du et al. (2022)	2022	Nude mice (BALB/c)	—	6	male	143Bcell	human	embryonic kidney cell	—	—
6	Wei et al. (2022)	2022	Nude mice (BALB/c)	—	4–6	—	MG-63cell	mice	BMSC	178.1	TSG101,CD81
7	Wang et al. (2022b)	2022	BALB/c nude mice	24	5	male	143Bcell	human	BMSC	30–200	CD63,Tsg101,Alix
8	Zhang et al. (2022)	2022	nude mice	32	4	—	MG-63cell	—	osteosarcoma cell	80–100	CD63,TSG101
9	Hu et al. (2023)	2023	Balb/c mice	28	4	female	K7M2cell	deer	antler stem cell	50–70	CD63,CD81,TSG101
10	Li et al. (2023)	2023	BALB/c nude mice	12	6–8	female	143Bcell	Pinctada martensii	mucilage cell	193.3	CD63,HSP70,CD9
11	Chen et al. (2023)	2023	BALB/c nude mice	68	5	male	143Bcell	human	BMSC	110	TSG101,CD9/63/81
12	Lu et al. (2024)	2024	BALB/c nude mice	12	4–6	female	HOS/MG 63cell	Dipsacus	Dipsacus cell	100–200	—

### 3.3 Bias risk assessment

In all, 25 studies reported baseline demographics of experimental mice, such as age, sex, and body weight, without significant bias. Six studies, meanwhile, reported placing animals at random during RCTs. We could not determine if researchers and/or animal handlers were blinded, whether animals were chosen randomly for outcome assessment, or whether researchers generated blinded results because of a lack of information. All of the papers selected reported the anticipated results despite the absence of a study protocol. All results from the offset risk evaluation of selected articles are summarized in Table 3.

**TABLE 3:** Quality assessment of studies according to SYRCLE.

	Items									
Study	D1	D2	D3	D4	D5	D6	D7	D8	D9	D10
Keremu et al. (2022)	-	+	-	-	-	-	-	+	+	+
Huang et al. (2022)	?	+	?	-	-	-	-	?	+	+
Du et al. (2022)	-	+	-	-	-	-	-	+	+	+
Yan et al. (2023)	-	?	-	-	-	-	-	?	+	+
Hu et al. (2023)	?	+	?	-	-	-	-	+	+	+
Wei et al. (2022)	-	?	-	-	-	-	-	?	+	+
Zhu et al. (2022)	?	+	?	+	-	-	-	+	+	+
Wang et al. (2022b)	?	+	?	-	-	-	-	+	+	+
Feng et al. (2022)	?	+	?	+	-	-	-	+	+	+
Lu et al. (2024)	?	+	?	-	-	-	-	+	+	+
Zhao et al. (2019)	?	+	?	-	-	-	-	+	+	+
Li et al. (2023)	?	+	?	-	-	-	-	+	+	+
Lu et al. (2022)	?	+	?	-	-	-	-	?	+	+
Zhang et al. (2022)	?	+	?	-	-	-	-	+	+	+
Zhan et al. (2022)	?	+	?	+	-	-	-	+	+	+
Chen et al. (2023)	-	+	-	+	-	-	-	+	+	+
Qi et al. (2021)	?	?	?	-	-	-	-	+	+	+
Li et al. (2021a)	?	+	?	-	-	-	-	+	+	+
Zhang et al. (2021)	-	-	-	-	-	-	-	+	+	+
Shi et al. (2021)	-	+	-	+	-	-	-	+	+	+
Li et al. (2021b)	-	-	-	-	-	-	-	+	+	+
Zhang et al. (2021)	-	-	-	-	-	-	-	+	+	+
Liu et al. (2021b)	-	+	-	+	-	-	-	+	+	+
Lin et al. (2019)	?	+	?	-	-	-	-	+	+	+
Xu et al. (2020b)	?	+	?	-	-	-	-	+	+	+

Risk of bias with SYRCLE tool. (A) Risk of bias graph. (B) Risk of bias summary. D1 (Selection bias): Was the allocation sequence adequately generated and applied? D2 (Selection bias): Were the groups similar at baseline, or were they adjusted for confounders in the analysis? D3 (Selection bias): Was the allocation adequately concealed? D4 (Performance bias): Were the animals randomly housed during the experiment? D5 (Performance bias): Were the caregivers and/or investigators blinded from knowledge of which intervention each animal received during the experiment? D6 (Detection bias): Were animals randomly selected for outcome assessment? D7 (Detection bias): Was the outcome assessor blinded? D8 (Attrition bias): Were incomplete outcome data adequately addressed? D9 (Reporting bias): Are reports of the study free of selective outcome reporting? D10 (Other): Was the study free of other problems that could result in a high risk of bias?

### 3.4 Meta-analysis results

**FIGURE 2:**
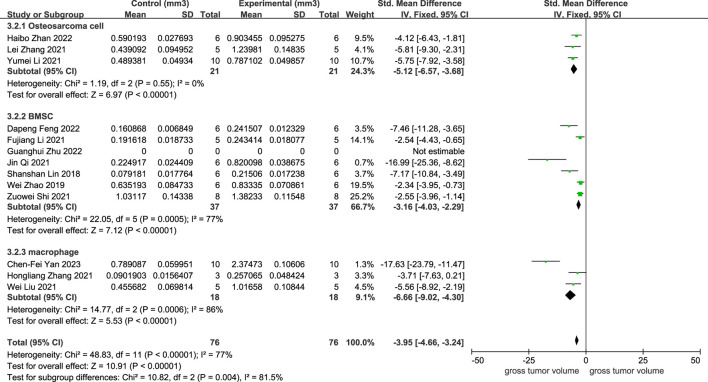
Meta-analysis results of studies on tumor volume increase after exosome transplantation.

For exosome-mediated positive and negative control of osteosarcoma progression, we examined 25 RCTs. Based on the several exosome sources (cell types), we performed a subclass analysis to investigate the acceleration of osteosarcoma development. Based on the several modes of action, we performed subclass analyses while investigating the suppression of osteosarcoma advancement. Our observations were as follows:

As illustrated in Figure 2, 12 studies revealed a rise in osteosarcoma volume following exosome transplantation, and the SMD value was −3.95 with a 95% confidence interval [-4.66, −3.24] under the fixed-effect model. At the same time, the heterogeneity test exhibited a P-value <0.00001, I2 = 77%. Subcategory evaluation revealed the following: the SMD value of OS cells was −5.12 with 95% confidence interval [-6.57, −3.68], the SMD value of BMSC cells was −3.16 with 95% confidence interval [-4.03, −2.29], and the SMD value of macrophage was −6.66 with 95% confidence interval [-9.02, −4.30].

**FIGURE 3:**
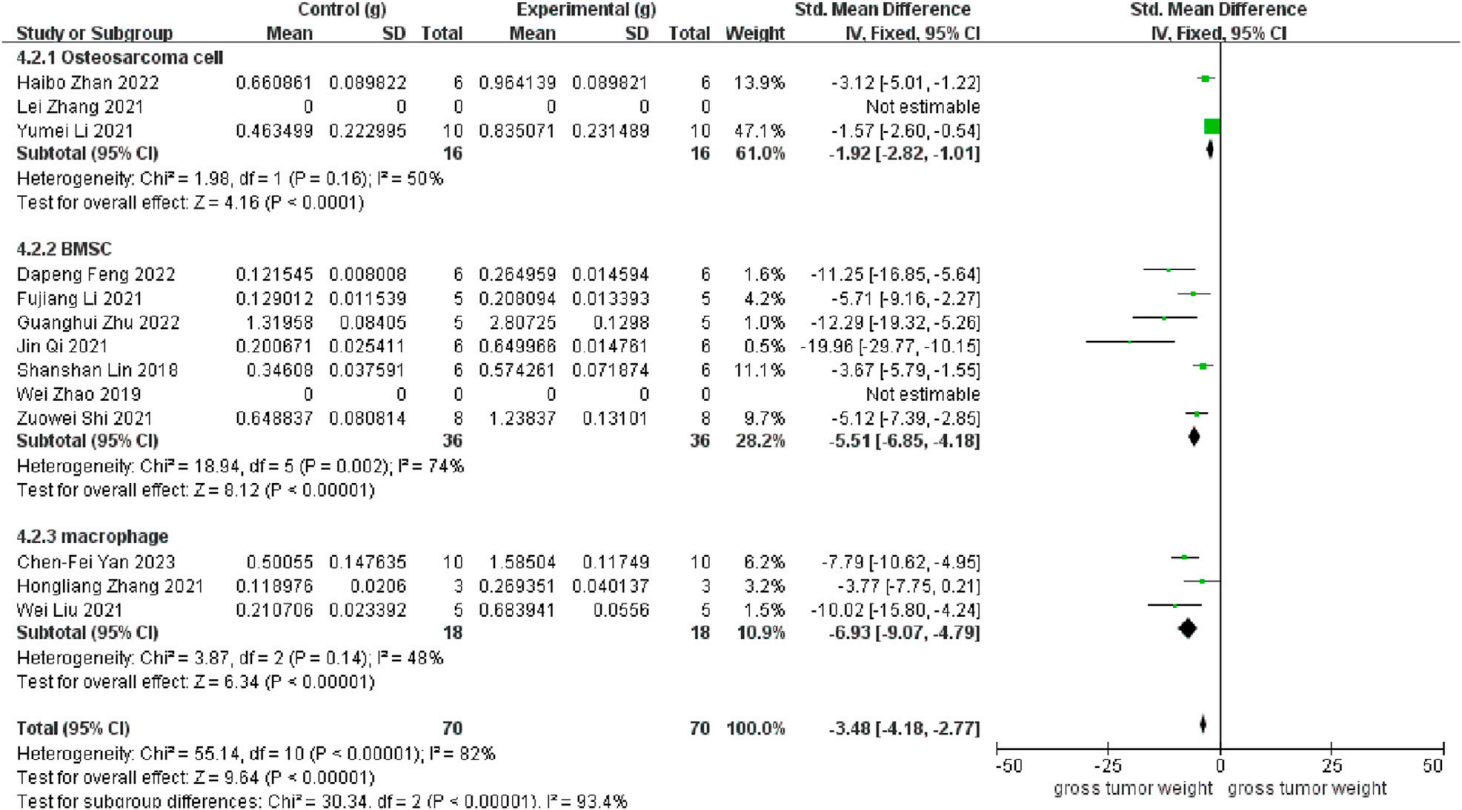
Meta-analysis results of studies on tumor weight increase after exosome transplantation.

As depicted in Figure 3, 11 studies revealed a rise in osteosarcoma weight following exosome transplantation, and the corresponding SMD value was −3.48 with 95% confidence interval [-4.18, −2.77] under the fixed-effect model, and the heterogeneity test exhibited a P-value <0.00001 and I2 = 82%. Subcategory evaluation revealed the following: the SMD value of OS cells was −1.92 with 95% confidence interval [-2.82, −1.01], the SMD value of BMSC cells was −5.51 with 95% confidence interval [-6.85, −4.18], and the SMD value of macrophage was −6.93 with 95% confidence interval [-9.07, −4.79].

**FIGURE 4:**
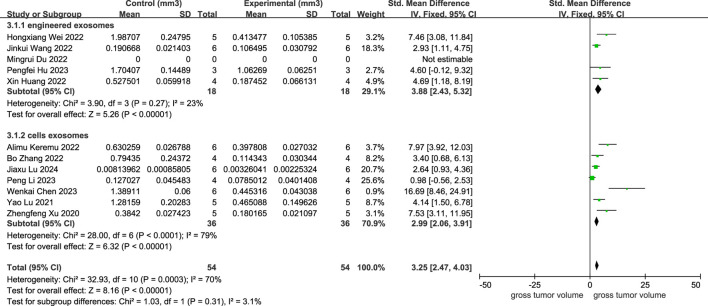
Meta-analysis results of studies on tumor volume decrease after exosome transplantation.

As provided in Figure 4, 11 studies depicted a decline in osteosarcoma volume following cellular exosome transplantation, and the corresponding SMD value was 3.25 with a 95% confidence interval [2.47, 4.03] under the fixed effect model, and the heterogeneity test exhibited a P-value equal to 0.0003, I2 = 70%. Based on the subcategory evaluation, the SMD value of engineered exosomes was 3.88 with a 95% confidence interval [2.43, 5.32], and the SMD value of cellular exosomes was 2.99 with a 95% confidence interval [2.06, 3.91].

**FIGURE 5:**
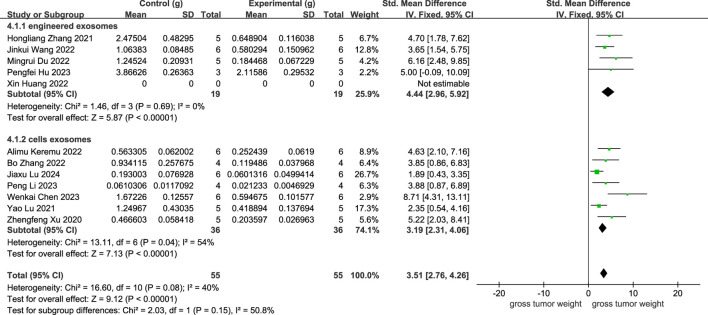
Meta-analysis results of studies on tumor weight decrease after exosome transplantation.

As revealed in Figure 5, 11 studies showed a rise in osteosarcoma weight following cellular exosome transplantation, with an SMD value of 3.51, 95% confidence interval of [2.76, 4.26], and a heterogeneity test of *p* = 0.08, I2 = 40% in the fixed-effects model. Subcategory analysis revealed the following: the SMD value of engineered exosomes was 4.44 with 95% confidence interval [2.96, 5.92], and the SMD value of cellular exosomes was 3.19 with 95% confidence interval [2.31, 4.06].

## 4 Discussion

**FIGURE 6:**
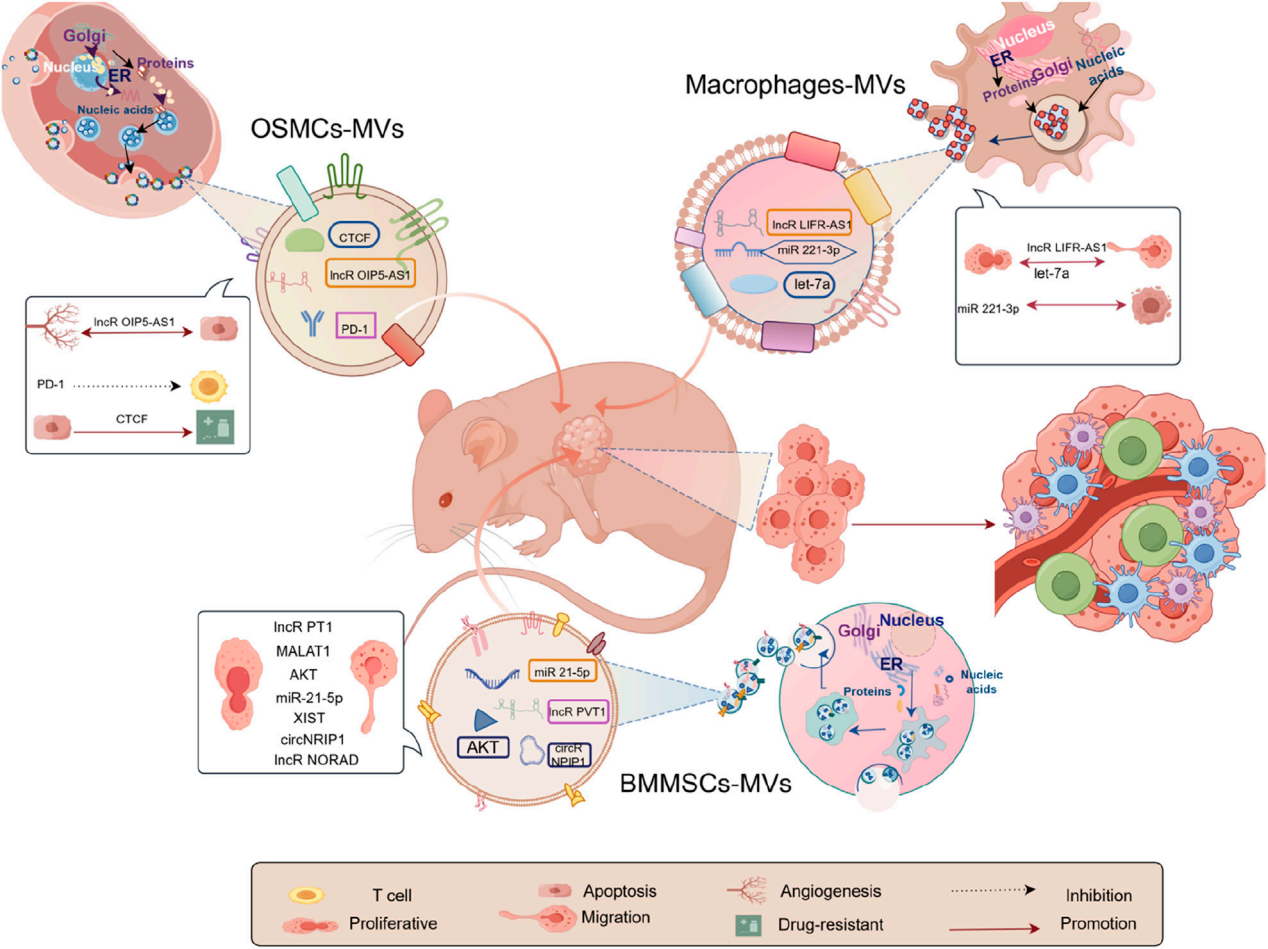
Pathogenesis of osteosarcoma due to exosomes.

We examined 25 RCTs investigating exosome transplantation’s effect on animal osteosarcoma models. The replies we received from our data differed based on the exosomal design. This suggests that exosomes have promise for exploring the pathophysiology of osteosarcoma and for driving the development of focused diagnostic methods. Firstly, exosomes can help us explore osteosarcoma pathogenesis. For example, long non-coding RNAs PVT1 (Zhao et al., 2019), miR-21–5p (Qi et al., 2021), CircNRIP1 (Shi et al., 2021), LncRNA XIST (Zhu et al., 2022), lncRNA NORAD (Feng et al., 2022) encapsulated by exosomes derived from bone marrow mesenchymal stem cells are now known. Macrophage-derived lncRNA Livr-AS1 (Zhang et al., 2021), miRNA-221–3p (Liu et al., 2021b), let-7a (Yan et al., 2023); lncRNA OIP5-AS1 (Li et al., 2021a) CTCF (Zhan et al., 2022) derived from osteosarcoma cells can all participate in the progression of osteosarcoma, as shown in Figure 6. As depicted in Figure 6, based on our meta-analysis of 13 studies, 3 categories of cellular exosomes regulated osteosarcoma pathogenesis in mice, and their origins were bone marrow mesenchymal stem cells (BMMSCs), osteosarcoma cells (OSMCs), and macrophages, respectively. While bone marrow mesenchymal stem cells (BMMSCs) were the most commonly studied exosomes, our comparative analysis revealed that macrophage exosomes exerted a substantially higher effect on the model mice than BMMSCs and osteosarcoma cells (OSMCs), in terms of both tumor volume and weight. Recent years have seen a rise in studies on macrophages and their derivatives, particularly tumor-associated macrophages (TAMs), crucial immune cells. They constitute a major component of the tumor microenvironments and play a key in regulating TME. TAMs also critically regulate tumourigenesis and progression (Duan and Luo, 2021; Chen et al., 2019). It has been reported that TAMs can be polarized into either pro-tumor (M2 macrophages) or anti-tumor phenotype (M1 macrophages) (Xu et al., 2020a). As expected, TAMs also regulate osteosarcoma development (Li et al., 2024; Wang et al., 2022a), solidifying the macrophage-based exosomal role in osteosarcoma without any exception. Moreover, several studies demonstrated the pathogenesis and progression of tumors via exosomes (Zhang et al., 2021; Liu et al., 2021b; Yan et al., 2023). Given this evidence, the analyzed studies depicted a vital role of macrophages in exosome development (from all three sources) in osteosarcoma (Li et al., 2024; Wang et al., 2022a). Further, they verified that macrophage exosomes play a critical role in the process (Zhang et al., 2021; Liu et al., 2021b; Yan et al., 2023). Therefore, while the experimental findings indicated that the exosomes from the 3 cells mentioned above types modulate osteosarcoma in model organisms, the authors hypothesize that macrophages exert a more significant influence on osteosarcoma progression and may thus hold greater promise for advancing research and therapeutic strategies in osteosarcoma. Therefore, the considerable function of macrophages and their derivatives in the pathogenesis of osteosarcoma necessitates additional investigation to establish a theoretical foundation for future study and the development of accurate and effective diagnostic and therapeutic strategies for osteosarcoma.

**FIGURE 7:**
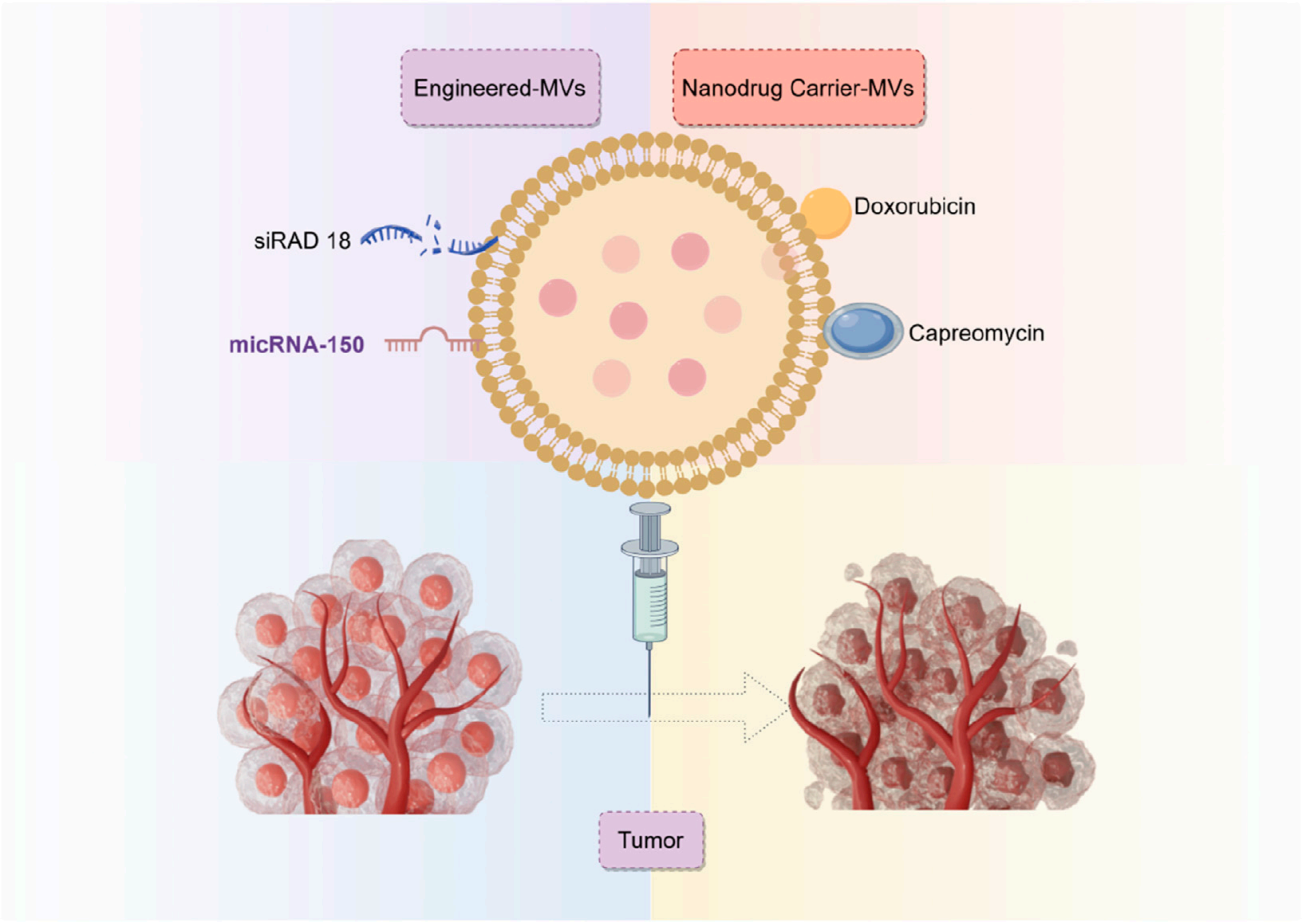
Mechanisms of exosome therapy for osteosarcoma.

Secondly, it is equally crucial to ascertain the potential of biological or engineered exosomes in the direct treatment of osteosarcoma or as an adjunct therapy. Exo-miR-150 targets IGF2BP1 (Xu et al., 2020b), Exo-miR-195 targets KIF4A (Lu et al., 2022), Exo-miR-206 targets NRSN2 (Keremu et al., 2022), and RGD-Exo targets Rad18 (Du et al., 2022), along with other pathways that impede the evolution of osteosarcoma. This significantly expands potential opportunities for innovation in diagnosing and treating osteosarcoma (Figure 7). It has shown excellent therapeutic effects not only in osteosarcoma exosomes but also in other malignant tumors, such as colon cancer, hepatocellular carcinoma, breast cancer, etc (Zhao et al., 2020).

Overall, 12 analyzed studies reported the role of exosomes in mouse models. Seven employed exosomes directly transplanted from humans, shellfish, and sequential cell sources to mouse models to serve as therapeutic agents. Our timeline analysis indicates that specialists have prioritized the importance of human-derived exosomes in osteosarcoma-related exosome research. Exosomes derived from animal and plant sources have been utilized as exosomal technologies advance and mature, producing favorable results. Furthermore, five projects, predominantly from 2022, examined the application of exosomes as carriers for targeted drug delivery by generating modified exosomes. Emerging evidence revealed that engineered exosomes possess properties such as easy mass production, excellent therapeutic efficacy, strong targeting, and not elicit drug resistance (Du et al., 2022; Huang et al., 2022; Wei et al., 2022; Wang et al., 2022; Hu et al., 2023). Ultimately, we performed a detailed assessment of two types of exosomes: those derived from transfected cells and those designed for drug delivery. We demonstrated that the drug-loaded exosomes exhibited enhanced therapeutic efficacy compared to exosomes derived from transfected cells. The comparison results validate the effectiveness of modified exosomes in osteosarcoma treatment, indicating that the ideal cellular exosomes for this purpose necessitate additional investigation. In summary, the authors warrant accelerating engineered exosomal design and development to fast-track its application in clinical settings. In the meantime, researchers should also explore other sources of exosomes to elucidate more suitable exosomes for osteosarcoma management.

Exosomes are an extracellular component facilitating substantial information interchange, providing exceptional diagnostic and prognostic capacities for osteosarcoma and its associated complications, in addition to their previously mentioned roles. In this meta-analysis, we examined pertinent and contemporary literature. Exosomes, including various biomarkers, serve diagnostic purposes (Araki et al., 2023; Han et al., 2021). In addition to the primary foci, Osteosarcoma is notorious for its enhanced metastatic potential. In this regard, it was reported that differences in exosome sources modulate the metastatic potential of osteosarcoma (Roberts et al., 2022). Lung metastasis is the most frequent metastatic foci of osteosarcoma, is a major contributor to cancer-related death, and has recently received extensive attention from experts and scholars. Numerous reports revealed that cellular exosomes are critical in modulating osteosarcoma lung metastasis (Deng et al., 2024; Yu et al., 2023; Chang et al., 2023; Almeida et al., 2022; Zhong et al., 2021; Zhang et al., 2021; Cheng et al., 2021). In addition to metastasis, alterations in bone metabolism are a major complication of osteosarcoma. Activation of osteoclast cell activity with simultaneous inhibition of osteogenesis promotes pathological fracture and pain among osteosarcoma patients, which contributes to severe physical and psychological suffering. As before mentioned, exosomes from osteosarcoma cells possess a robust information interaction ability, which induces the formation of bone metabolic abnormalities within osteosarcoma patients (Ucci et al., 2021; Luo et al., 2021; Raimondi et al., 2020). Likewise, engineered exosomes promote bone formation in the osteosarcoma pathological state, thereby preventing bone destruction-mediated complications via precise targeting action (Gupta et al., 2023). Finally, exosomes also predict drug resistance, prognosis, and survival of osteosarcoma patients (Araki et al., 2023; Weinman et al., 2021; Wang et al., 2021; Wang et al., 2023).

We confirmed the crucial role of exosomes in the progression of osteosarcoma using meta-analysis and systematic review. We also found that exosomes generated from macrophages exceeded other sources in osteosarcoma regulation. Secondly, we identified the incredible potential of exosomes in osteosarcoma treatment and determined the efficacy of engineered exosomes and future research direction involving cellular exosomes based on our subcategory analysis. To expand our understanding of exosomes in osteosarcoma pathogenesis, progression, and treatment, we examined recent articles about the association between osteosarcoma and exosomes and osteosarcoma and related complication diagnostic and prognostic prediction.

### 4.1 Study strengths and weaknesses

Strengths of this study: (1) We were the first to extensively evaluate the significance of exosomes on osteosarcoma pathogenesis and in interventional therapy. We summarized the overall progression of the study and highlighted the challenges and the direction of future development. (2) Our hypotheses were entirely validated by a thorough exosome feature-based subcategory analysis that we created. Furthermore, we developed a solid research hypothesis for additional studies based on our subcategory analysis findings. (3) We reviewed recent investigations involving exosomes and osteosarcoma diagnosis and prognostic prediction, summarized overall research progress, and provided the basis for expanding ideas of related studies. (4) Using the SYRCLE risk of bias assessment method, we evaluated the intrinsic risk of bias in the investigated studies. Furthermore, we limited our selection to the most current findings from the same research organization within 5 years to reduce bias in those studies.

Limitations of this study: (1) Data was selected based on tumor size and weight in mouse models. We did not analyze other outcome indicators. This could lead to potential bias. Therefore, the reliability of our model requires further validation. (2) We could not accurately identify the heterogeneity source; thus, we employed SMD for data merging analysis, which produced more conservative conclusions. (3) We did not review grey literature and conference abstracts and opted for English-only databases, which may have introduced publication bias. (4) The quantity of examined studies and associated sample population were relatively small, potentially generating bias. (5) All included studies had an unclear blinding performance bias risk.

## Data Availability

The original contributions presented in the study are included in the article/supplementary material, further inquiries can be directed to the corresponding author.
